# Supplementary material for: COZOID: contact zone identifier for visual analysis of protein-protein interactions
Source: BMC Bioinformatics. 2018 Apr 6;19:125. doi: 10.1186/s12859-018-2113-6 (PMC5889581; doi:10.1186/s12859-018-2113-6)
Supplement: Supplementary file 2 — Software build. Executable binary file of the software tool. (ZIP 118,784 kb) [file 12859_2018_2113_MOESM2_ESM.zip › cozoid/platform/docs/testng-6.5.1-javadoc.zip!/org/testng/package-frame.html]

org.testng (TestNG)


org.testng

Interfaces 
  
*IAnnotationTransformer*
  
*IAnnotationTransformer2*
  
*IAttributes*
  
*IClass*
  
*IConfigurable*
  
*IConfigurationListener*
  
*IConfigurationListener2*
  
*IConfigureCallBack*
  
*IExecutionListener*
  
*IExtraOutput*
  
*IHookable*
  
*IHookCallBack*
  
*IInstanceInfo*
  
*IInvokedMethod*
  
*IInvokedMethodListener*
  
*IInvokedMethodListener2*
  
*IMethodInstance*
  
*IMethodInterceptor*
  
*IMethodSelector*
  
*IMethodSelectorContext*
  
*IModuleFactory*
  
*IObjectFactory*
  
*IObjectFactory2*
  
*IReporter*
  
*IResultMap*
  
*IRetryAnalyzer*
  
*ISuite*
  
*ISuiteListener*
  
*ISuiteResult*
  
*ITest*
  
*ITestClass*
  
*ITestClassFinder*
  
*ITestContext*
  
*ITestListener*
  
*ITestMethodFinder*
  
*ITestNGListener*
  
*ITestNGListenerFactory*
  
*ITestNGMethod*
  
*ITestObjectFactory*
  
*ITestResult*
  
*ITestRunnerFactory*

Classes 
  
Assert
  
AssertJUnit
  
ClassMethodMap
  
CommandLineArgs
  
ConversionUtils
  
Converter
  
DependencyMap
  
FileAssert
  
Reporter
  
ReporterConfig
  
ReporterConfig.Property
  
SuiteRunner
  
SuiteRunnerWorker
  
SuiteRunState
  
TestListenerAdapter
  
TestNG
  
TestNG.ExitCodeListener
  
TestNGAntTask
  
TestNGAntTask.TestNGLogSH
  
TestNGUtils
  
TestRunner

Enums 
  
TestNGAntTask.Mode

Exceptions 
  
SkipException
  
TestException
  
TestNGException
  
TimeBombSkipException
